# The RIP1–RIP3 Complex Mediates Osteocyte Necroptosis after Ovariectomy in Rats

**DOI:** 10.1371/journal.pone.0150805

**Published:** 2016-03-17

**Authors:** Hongwang Cui, Yongjun Zhu, Dianming Jiang

**Affiliations:** 1 Department of Orthopaedics, The First Affiliated Hospital of Chongqing Medical University, Chongqing, China; 2 Department of Nephrology, The First Affiliated Hospital of Chongqing Medical University, Chongqing, China; Nanjing Medical University, CHINA

## Abstract

Osteocyte apoptosis has been reported to play a central role in bone remodeling. In addition to apoptosis, other mechanisms may be involved in osteocyte loss. This study aimed to investigate the effect of necroptosis on osteocytes in ovariectomized (OVX) rats. Ninety-six female Sprague-Dawley rats were randomly divided into an OVX group and a sham group. At 0, 4, 8 and 12 weeks after surgery, specimens from each group (n = 12 each) were harvested. Bone mineral density (BMD) and body weight were measured. Transmission electron microscopy (TEM) and micro-CT were used to observe the changes in cellular morphology and bone microarchitecture induced by estrogen deficiency. Osteocyte apoptosis and necroptosis were evaluated via TUNEL and immunofluorescence staining for active *caspase-3*. At 8 weeks after ovariectomy, a greater number of osteocytes with typical necrotic morphological features were TUNEL positive but negative for active caspase-3. Western blotting, quantitative real-time PCR and immunofluorescence assessments demonstrated that the levels of receptor-interacting serine/threonine protein kinase 1 (RIP1) and RIP3 in osteocytes were significantly increased at 8 weeks after ovariectomy. These data are the first to suggest that necroptosis accelerates osteocyte loss under conditions of estrogen deficiency-induced osteoporosis in OVX rats. These findings provide evidence of a potential mechanism through which osteocyte necroptosis is associated with postmenopausal osteoporosis.

## Introduction

Low bone mass and microarchitectural deterioration, which lead to increased bone fragility and greater susceptibility to fracture, are the main characteristics of osteoporosis. Bone metabolism in women is regulated in part by estrogen. Postmenopausal osteoporosis is one of the main forms of osteoporosis and occurs in women after natural or surgically induced menopause. Osteoblast and osteocyte apoptosis that takes place in response to estrogen deficiency is an important factor in postmenopausal osteoporosis [1].

Ovariectomized (OVX) rats are a widely used model [2,3]for estrogen deficiency-induced bone loss. The studies using this model have examined the short-term effects of cytokines and hormones[4].Previous studies have demonstrated that increased osteocyte apoptosis occurs within 4 weeks after estrogen withdrawal[5–8]. Measurement of the response to OVX via dual-energy x-ray absorptiometry (DXA) revealed a statistically significant decrease in bone mineral density (BMD) compared with sham controls as early as 12-16weeks post-OVX[9]. Unfortunately, following OVX for 4 weeks, the time-related changes in osteocytes have not been observed as the duration of estrogen withdrawal increases. Thus, the time-related changes in osteocytes as the duration of estrogen withdrawal increases must be carefully elucidated.

In addition to apoptosis, there may be numerous other mechanisms for regulating cell death that participate in osteocyte loss. A notable example is necroptosis, a form of caspase-independent programmed cell death associated with morphological changes similar to those that occur in necrosis. Receptor-interacting serine/threonine protein kinases 1 and 3 (RIP1/3) play vital roles in the necroptotic pathway[10–12].It has been confirmed that necroptosis is an important pathological phenomenon involved in glucocorticoid-induced osteoporosis[13].However, the role of necroptosis in osteocyte loss in OVX rats is currently unknown. In this study, an OVX rat model was used to determine whether estrogen deficiency leads to increased osteocyte necroptosis, and we investigated the expression of RIP1 and RIP3 in OVX rats.

## Materials and Methods

### Animals and experimental design

Sixteen-week-old healthy female Sprague-Dawley rats (from the animal laboratory center of Chongqing Medical University) were used in this study. All of the rats were housed in cages under standard laboratory conditions with a 12 hours light/dark cycle, with free access to water and a standard rodent diet. After a 7-day adaptation period, 96rats were randomly assigned to the OVX group or the sham group. Forty-eight animals were subjected to bilateral OVX under pentobarbital sodium anesthesia. The remaining 48 animals underwent sham operation, in which the ovaries were exteriorized and then replaced in the abdominal cavity. After surgery, the animals were allowed unrestricted cage activity and ad libitum access to food and water. During the experiment, there were no unintended death, and all of the rats exhibited good health and well-being. All of the protocols were approved by the Division of Laboratory and Animal Medicine of Chongqing Medical University for Medical Sciences.

### Sample harvest and preparation

The animals were weighed at the beginning and end of the experiment. At 0, 4, 8 and 12 weeks after surgery, the bilateral proximal femoral BMD was determined via DXA. At various time points after surgery, the rats in the OVX and sham groups (n = 12rats/group) were anesthetized as described above, and blood samples were collected through cardiac puncture, after which the animals were euthanized via cervical dislocation. The blood samples were centrifuged to separate the plasma. Serum was stored at −80°C for subsequent biochemical measurements. After the animals were sacrificed, both the right and left proximal femurs (5 mm distal and proximal to Ward’s triangle) were dissected, and the soft tissue was removed from these bones. The bone marrow was removed from the proximal femur through repeated washes with phosphate-buffered saline (PBS). The right femurs of each group were briefly immersed in liquid nitrogen for subsequent qPCR (n = 6rats/group) and Western blot (n = 6rats/group) analyses. Six of the left femurs from each group were fixed in10% neutral buffered formalin for 48 hours at 4°C for micro-CT analysis. Following micro-CT measurements, the left femurs were decalcified in 15% ethylenediaminetetraacetic acid (EDTA, pH7.4) for 4 weeks for immunofluorescence analysis. The other six left femurs were fixed in 2.5% glutaraldehyde for 24 hours at 4°Cand decalcified in 15% EDTA (pH 7.4) for 2 weeks prior to transmission electron microscopy (TEM) analysis.

### Measurement of collagen type I cross-linked C-telopeptide (CTX)

An enzyme-linked immunosorbent assay (ELISA) was performed to measure the levels of CTX, a bone resorption marker, in rat serum (Uscn Life Science Inc, Wuhan, china) according to the manufacturer’s protocol.

### Bone mineral density measurements

Areal BMD was measured via DXA (Hologic Discovery, Bedford, MA, USA).The rats were anesthetized with pentobarbital sodium and placed in a horizontal position. The bilateral proximal femoral BMD (S1 Fig) was scanned (pixel area resolution: 640 mm^2^).The scans were analyzed using specialized software for small animals supplied by the equipment's manufacturer.

### Micro-CT measurements

Ward's triangle is the region of the intersection of the compressive and tensile trabecular systems and the concentrated stress site for the cancellous bone within the femoral neck region, as well as the key area of osteoporosis of the femoral neck(S2 Fig)[14]. To analyze the 3D microarchitecture of the bone, Ward’s triangle was scanned with a micro-CT system (viva CT40, SCANCO Medical AG, Zürich, Switzerland). The volume of interest(VOI, 29 × 29 × 29μm3) was selected using a semiautomatic contouring method. The scans were performed with the following settings: 15 μm resolution, 70kVp, 114 μA, and 250ms integration time. A series of planar transverse gray-value images was generated to reconstruct 3Dimages. Micro-architectural parameters within the VOI, including the bone volume over the total volume (BV/TV), trabecular thickness (Tb.Th), trabecular number (Tb.N), and trabecular separation (Tb.Sp), were calculated automatically using the micro-CT system software.

### Transmission electron microscopy

Specimens of 1mm^3^ from Ward’s triangle were fixed in 2.5%glutaraldehyde for 24 hours at 4°C and then washed three times with 0.1 M PBS (pH 7.4). Next, the specimens were demineralized in15% EDTA for 2 weeks at room temperature. After three washes with 0.1 M PBS, the tissue fragments were fixed in 2% osmium tetroxide for 1 hour and block-stained with 2% uranyl acetate. Next, the bone tissues were embedded in epoxy resin and dehydrated. Sections (80nm) were cut and stained with uranyl acetate and lead citrate. The ultrastructure of the bone tissue was observed via TEM (Hitachi-7500, Hitachi, Tokyo, Japan).

### Immunofluorescence detection of cleaved *caspase-3* and in situ fluorescence TUNEL staining

The proximal femur was fixed in 10% neutral buffered formalin for 48 hours at 4°C and decalcified in 15% EDTA at room temperature for 4weeks. Sagittal tissue sections (4μm thick) were prepared for immunofluorescent and in situ fluorescent TUNEL staining. For antigen retrieval, the sections were incubated with 20 μg/ml proteinase K for 15 minutes at37°C after three washes with 0.1 MPBS. After treatment with 0.1%Triton X-100, the sections were blocked with 10% goat serum, incubated with a rabbit polyclonal antibody against cleaved caspase-3 (Cell Signaling Technologies, Danvers, MA, USA;1:100 dilution) overnight at 4°C, and then incubated with Alexa Fluor 594-conjugated goat anti-rabbit IgG (Beyotime, Nantong, Jiangsu, China). After three washes with 0.1 MPBS, the sections were assayed using an in situ cell death detection kit (Roche, Basel, Switzerland) according to the manufacturer’s instructions. All of the sections were counterstained with 4', 6-diamidino-2-phenylindole (DAPI). Finally, the number of total cells, TUNEL-positive cells, with and without cleaved caspase-3-positive cells were counted in three to five non-contiguous high power fields for each specimen using a laser scanning confocal microscope (LEICA TCS SP2, Wetzlar, Germany), with and without these values were used to calculate the percentages of TUNEL-positive cells and cleaved caspase-3-positive cells. Cell counting was performed by a pathologist blinded to the experimental conditions.

### Double immunofluorescent staining for *RIP1* and *RIP3*

Tissue sections were treated as described above. After treatment with 0.1%Triton X-100, the sections were blocked with 10% goat serum and incubated overnight at 4°C with a mouse RIP1 monoclonal antibody (Abcam, Cambridge, MA, USA; 1:100 dilution) and a rabbit RIP3 polyclonal antibody (Abcam, Cambridge, MA, USA; 1:100 dilution), followed by incubation with Alexa Fluor 594-conjugatedgoat anti-mouse IgG and Alexa Fluor 488-conjugatedgoat anti-rabbit IgG (Beyotime, Nantong, Jiangsu, China). After three washes with 0.1 MPBS, the sections were counterstained with DAPI. Finally, the numbers of total cells and RIP1/-RIP3-positive cells were determined using a laser scanning confocal microscope (LEICA TCS SP2, Wetzlar, Germany).

### Quantitative real-time PCR (qPCR)

Total RNA was extracted from the proximal femur using RNAiso Plus (Total RNA Extraction Reagent, TaKaRa Bio Inc, Tokyo, Japan) in accordance with the manufacturer’s protocol. Approximately 1,000ng of total RNA was reverse transcribed using the Prime Script® TM RT Reagent Kit with gDNA Erase (TaKaRa Bio Inc, Tokyo, Japan). qPCR was performed in triplicate in a 20μl volume, using SYBR® Premix Ex Taq^TM^ II (TaKaRa Bio Inc, Tokyo, Japan) and the CFX96 Touch^TM^ Real-Time PCR Detection System (Bio-Rad, California, USA) according to the manufacturers’ instructions. The following primer sequences were used: RIP1 forward, 5'-AGGTACAGGAGTTTGGTATGGGC-3', and reverse, 5'-GGTGGTGCCAAGGAGATGTATG-3'; RIP3 forward, 5'-TAGTTTATGAAATGCTGGACCGC-3', and reverse, 5'-GCCAAGGTGTCAGATGATGTCC-3'; and GAPDH forward, 5'-AGTTCAACGGCACAGTCAAGG-3', and reverse, 5'-TCACCCCATTTGATGTTAGCG-3'. Gene expression was determined relative to the housekeeping gene GAPDH using the 2^-ΔΔCt^ method.

### Western blotting

The proximal femur specimens frozen in liquid nitrogen were placed in a mortar for pulverization, followed by homogenization in ice-cold radio immunoprecipitation assay (RIPA) buffer (Beyotime, Nantong, Jiangsu, China).The total protein concentration was determined using a BCA Protein Assay kit (Beyotime, Nantong, Jiangsu, China) according to the manufacturer’s instructions. Samples containing approximately 80 μg of protein were separated via SDS-PAGE, and the proteins were transferred to a PVDF membrane (EMD Millipore, Billerica, Massachusetts, USA). After blocking non-specific binding sites with a 5%non-fat dry milk solution for 1.5 hours at room temperature, the membrane was incubated overnight at 4°C with the following primary antibodies: anti-RIP1 monoclonal antibody (Abcam, Cambridge, MA, USA), anti-RIP3 polyclonal antibody (Abcam, Cambridge, MA, USA) and anti-β-actin monoclonal antibody (Santa Cruz Biotechnology, CA, USA). Western blotting was performed using conventional methods, as described previously [15]. The protein bands of interest were visualized using an ECL detection kit (Beyotime, Nantong, Jiangsu, China). Protein expression was quantified via densitometry analysis using Image Lab version 2.1 (Bio-Rad). The quantitative densitometric values for each protein were normalized to those of β-actin.

### Statistical analysis

Data are presented as the mean± standard error of the mean (SEM). Statistical analyses were performed with SPSS software(version 17.0). At different time points, significant differences in the OVX groups were evaluated through ANOVA, followed by a least significant difference (LSD) or Dunnett’s post-hoc test. Mann-Whitney test was used for comparisons between the sham and OVX groups at the same time point. P<0.05 was considered statistically significant.

## Results

### Ovariectomy induces bone loss

#### Body weight

**Fig 1:**
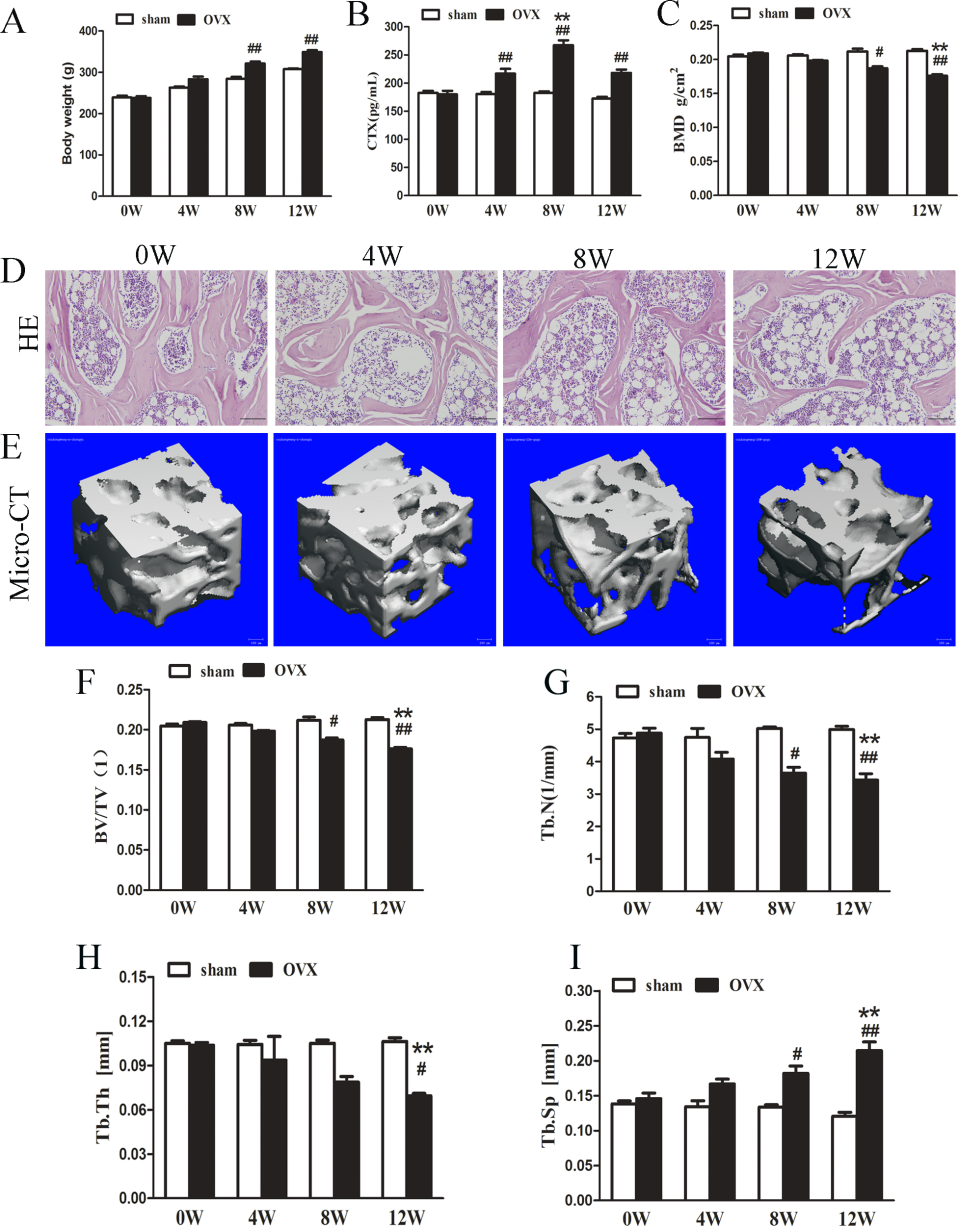
Effect of ovariectomy on rats. (A) Changes in body weight. (B) Changes in serum CTX. (C) Changes in BMD in the proximal femur. (D) Representative photomicrographs of H&E-stained sections. The photomicrographs were obtained from OVX rats at various time points. Scale bars represent 25 μm. (E) Micro-CT evaluation of Ward’s triangle. Representative 3D reconstructed images were obtained from OVX rats at various time points. Scale bars represent 100μm. (F) BV/TV. (G) Tb.Th. (H) Tb.N. (I) Tb.Sp. The data are presented as the mean ± SEM (n = 6rats/group). #p<0.05, ##p<0.01 vs the sham group at the same time point; *p<0.05, **p<0.01 vs the OVX group at 0 weeks. Sham, sham-operated group. OVX, ovariectomy group.W,week. BMD, bone mineral density; BV/TV, bone volumefraction; Tb.Th, trabecular thickness; Tb.N, trabecular number; Tb.Sp, trabecular separation.

It has been reported that OVX results in weight gain[16,17]. In this study, animal weight was measured, and the average values are shown in Fig 1A.The body weight of the animals was higher in the OVX group than in the sham group at two time points (8, and12weeks).

#### Effect on biochemical bone markers

The serum levels of biomarkers of bone turnover were measured as indicators of the effects of OVX on bone remodeling. Serum CTX levels, an accepted marker of bone resorption, were increased in the OVX rats compared with the sham-operated rats at different time points (4, 8, and 12weeks). Furthermore, CTX levels were significantly increased in the OVX group at 8 weeks compared with0 week (Fig 1B).

#### Bone mineral density measurements

BMD of the proximal femur was determined through DXA analysis to be significantly reduced in the OVX rats at 12 weeks compared with at 0 week; however, there were no significant differences in the sham groups at all time points (Fig 1C).

#### Histological assessment

Hematoxylin and eosin (H&E) staining was performed to assess the number of bone trabeculae in Ward’s triangle. H&E staining showed increased interruptions and separation of the trabecular bone network, as well as a reduced trabecular bone mass in Ward’s triangle over time in the OVX group (Fig 1D).

#### Micro-CT assessment

Micro-CT was performed to assess the bone microarchitecture in Ward’s triangle (Fig 1E). At 12weeks, a 3D reconstruction of Ward’s triangle showed that BV/TV (Fig 1F), Tb.N (Fig 1G) and Tb.Th (Fig 1H) were significantly decreased in the OVX group compared with the sham group. Tb.Sp in the OVX group achieved a significantly greater than those of the sham group (Fig 1I). The above microarchitectural parameters, with the exception of Tb.Th, were significantly different in the OVX group compared with the sham group at 8 weeks. These parameters became progressively more pronounced with time up to 12 weeks after OVX. However, the microarchitectural parameters were no significant difference in the sham-operated rats at different time points.

In summary, these results provide evidence that OVX rats were an appropriate animal model of estrogen withdrawal-induced bone loss. These results are consistent with previous reports [2,18,19].

## Histological detection of osteocyte necroptosis

**Fig 2:**
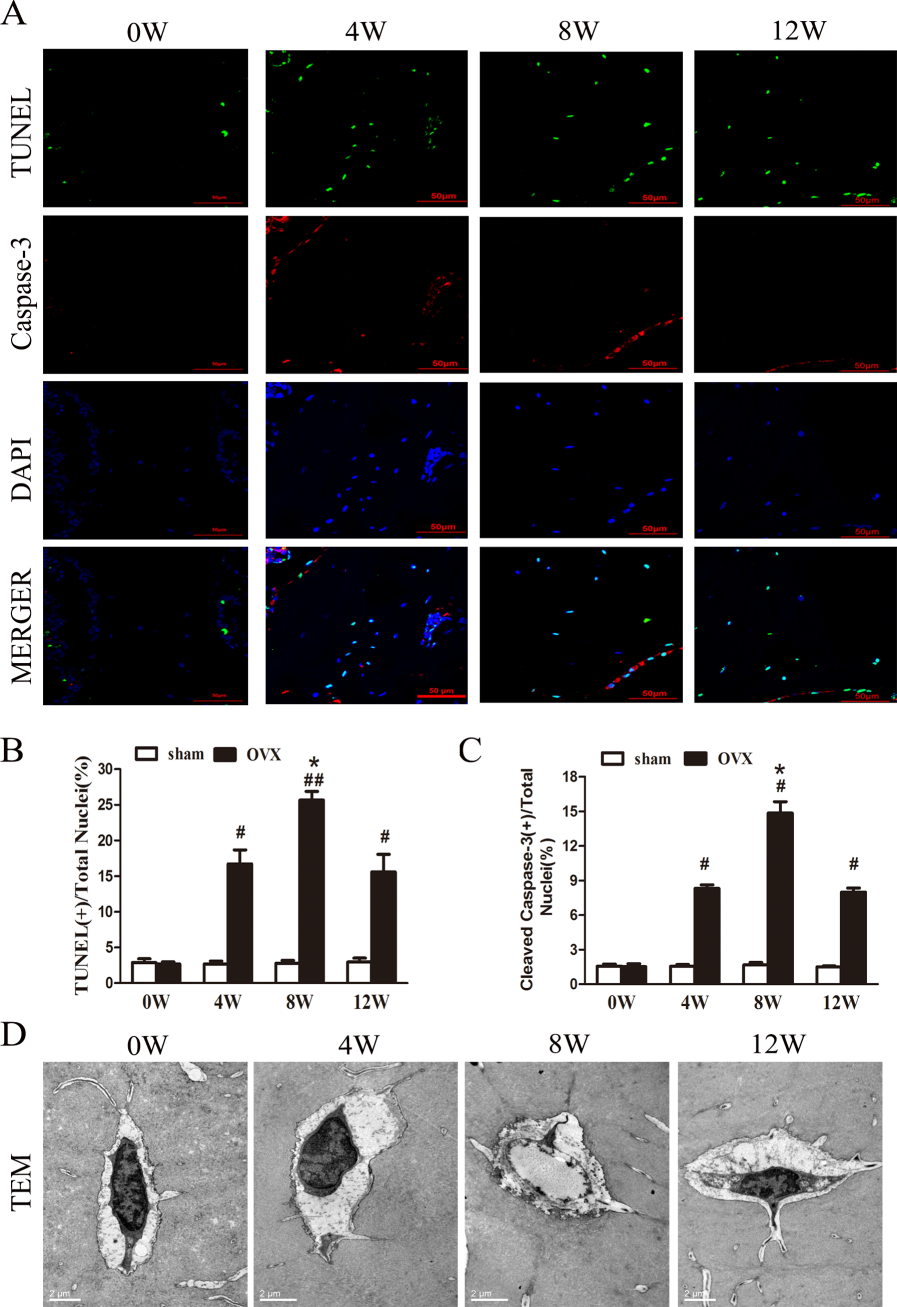
Effect of ovariectomy on osteocytes. (A) TUNEL-stained (green fluorescence) cells in decalcified bone were co-immunofluorescence staining to detect caspase-3 (red fluorescence) and the nucleus (DAPI, blue fluorescence). Scale bars represent50μm. (B) The data are presented as the % ratio of TUNEL-positive cells. (C) The data are presented as the % ratio of cleaved caspase-3-positive cells. (D) The changes in osteocytes from rats in different OVX groups were visualized via TEM. Scale bars represent 2 μm. Necroptotic cells with typical necrotic morphological features were observed via TEM in Ward’s triangle at 8 weeks after OVX; the data are presented as the mean ± SEM (n = 6rats/group). #p<0.05, ##p<0.01 vs the sham group at the same time point; *p<0.05, **p<0.01 vs the OVX group at 0 weeks. Sham, sham-operated group. OVX, ovariectomy group. W, week.

TUNEL and anti-active caspase-3 staining are often used to determine the type of cell death. Typically, cells that are positive for TUNEL staining but negative for active caspase-3 are considered necrotic cells [20–22]. In this study, the apoptotic cells in decalcified bone were visualized through positive TUNEL (green fluorescence) and caspase-3 (red fluorescence) immunofluorescence staining and nuclear staining (DAPI, blue fluorescence) (Fig 2A). The percentage of TUNEL-positive osteocytes is displayed in Fig 2B. The percentage of TUNEL-positive cells was significantly higher in the OVX rats at 8 weeks than at 0 week (p < 0.05). An interesting phenomenon was observed: active caspase-3 staining occurred predominantly in osteoblasts and was absent in osteocytes (Fig 2A). The number of activecaspase-3-positive osteocytes was significantly higher in the OVX rats at 8 weeks than at 0 weeks (Fig 2C). Furthermore, some of the osteocytes were TUNEL positive but lacked staining for active caspase-3 (Fig 2A).

To better understand the morphological characteristics of the bone cells of OVX rats, we performed TEM to observe the ultrastructure of the bone. Fig 2D shows the microstructural changes observed in osteocyte via TEM. Following OVX for 8 weeks, we recorded an interesting phenomenon that most of the osteocytes displayed a necrotic morphology, with marked swelling, along with membranolysis, disappearance of organelles and extensive formation of intracellular vacuoles. These findings were consistent with the typical morphological features of necroptotic cell death [10,13,23,24].

### *RIP1* and *RIP3* expression in osteocytes

**Fig 3:**
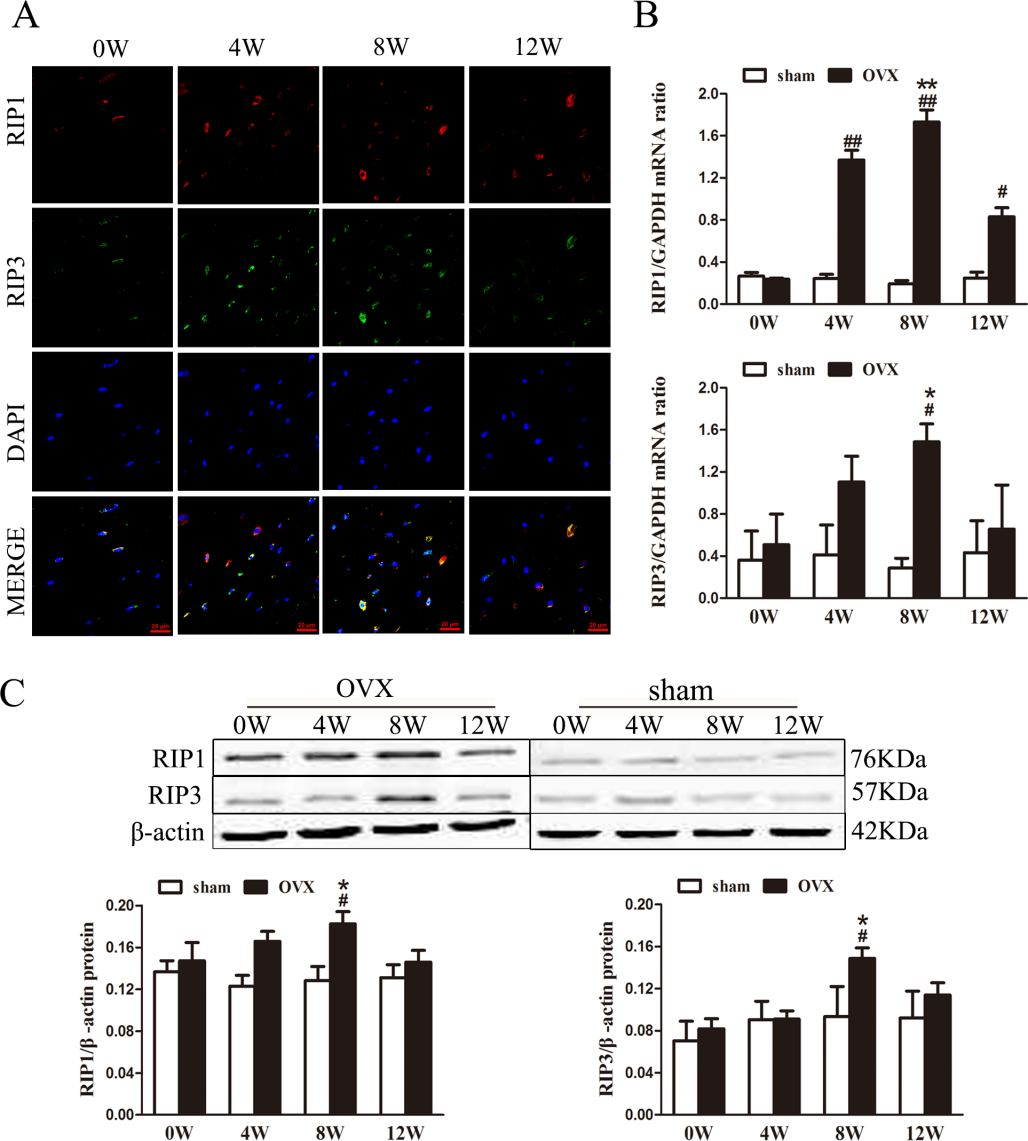
Effect of ovariectomy on *RIP1* and *RIP3* expression in osteocytes. (A) Representative photomicrographs of decalcified bone sections stained to detect RIP1 (red fluorescence), RIP3 (green fluorescence), and the nucleus (DAPI, blue fluorescence). Scale bars represent 20μm. (B) Quantitative real-time PCR analysis of RIP1 and RIP3 mRNA expression (n = 6rats/group). (C) Western blot analysis of RIP1 and RIP3 protein expression (n = 6rats/group). The data are presented as the mean ± SEM (n = 6rats/group). #p<0.05, ##p<0.01 vs the sham group at the same time point; *p<0.05, **p<0.01 vs the OVX group at 0 weeks. Sham, sham-operated group. OVX, ovariectomy group. W, week.

We studied the localization of RIP1 and RIP3, which are critical bio-markers of necroptosis [10,23], in osteocytes via immunofluorescence. Under confocal microscopy, we observed that RIP1 colocalized with RIP3 in some osteocytes from OVX rats at 8weeks, especially in the osteocytes cytoplasm (Fig 3A).

RIP1 and RIP3mRNA expression was significantly higher in OVX rats at 8 weeks than at 0 week (Fig 3B). However, there were no significant differences detected in the sham groups. These results from the western blot analysis were consistent with the results from RIP1 and RIP3 mRNA analysis (Fig 3C).

## Discussion

Postmenopausal osteoporosis, the most widespread metabolic bone disease, occurs after natural or surgically induced menopause and is associated with a decrease in bone mass and deterioration of the trabecular architecture [25]. The imbalance between bone formation and bone resorption is considered a key pathological mechanism in osteoporosis. Osteocyte apoptosis plays an important role in the development of osteoporosis [7]. Previous studies have demonstrated that increased osteocyte apoptosis occurs after estrogen withdrawal [1,5]. Osteocytes may therefore play an important role in regulating both bone resorption and bone formation [26]. Emerton found that estrogen withdrawal in ovariectomized mice results in osteocyte apoptosis in the femoral cortex [8]. Consistent with previous publications [5,7,8], the loss of estrogen in OVX rats caused an increase in osteocyte apoptosis in this study. TUNEL is a classical method for detecting of DNA fragmentation [27]; this technique has long been considered the gold standard for detecting apoptosis in situ. However, false-positive TUNEL results may arise after necrotic cell death [28]. We found that some osteocytes were TUNEL positive, but negative for active caspase-3, suggesting that there may be another mechanism besides to apoptosis, that is involved in osteocyte loss. In contrast to apoptosis, cell death via necrosis occurs mainly as a result of disrupted membrane integrity and cellular swelling to the point of bursting. In fact, we utilized TEM to determine that most of the osteocytes in OVX rats had undergone necrotic cell death. All of these data led us to consider that another cell death mechanism might affect osteocyte survival under conditions of estrogen deficiency.

Necroptosis is a highly regulated caspase-independent form of programmed cell death with morphological similarities to necrosis and is stimulated by oxidative stress, tumor necrosis factors, and toll-like receptor activation [29]. The complete mechanism underlying necroptosis remains unclear, but RIP1 with RIP3 plays vital roles in the necroptosis pathway [11,30,31]. Upon the induction of necroptosis, RIP3 is recruited to RIP1 to establish a protein complex that initiates necroptosis. It is believed that osteocytes play a role in bone loss in osteoporosis [32].However, whether the necroptosis pathway contributes to osteocyte cell death in OVX rats is currently unknown. In this study, to investigate whether cell death was induced by necroptosis in OVX rats, RIP1 and RIP3 expression levels were measured in tissues via western blot analysis and qPCR, and RIP1/RIP3 colocalization assays were performed [22]. The levels of RIP1 and RIP3 peaked at 8weeks after OVX, and necroptotic cells with typical morphological features were observed through TEM at the same point. Furthermore, at 8 weeks post-OVX, RIP1 colocalized with RIP3. These results indicated that necroptosis participated in the bone loss in the OVX rats. The findings of the present study confirm the role of estrogen in the osteocyte viability regulation and indicate new field of preventing osteoporosis.

Most studies have demonstrated that increased osteocyte apoptosis occurs within 4 weeks after estrogen withdrawal [6–8,33]. The cortical bone of ovariectomized rats exhibits a 4–7-foldincrease in the number of apoptotic osteocytes, as detected by active caspase-3 immunostaining. Interestingly, the apoptotic osteocytes are mainly located in the posterior portion of the diaphyseal cortex. The increased numbers of apoptotic osteocytes in the posterior femoral cortex were seen by day 3 after OVX, and decreased slowly thereafter. Osteocyte apoptosis remained increased at 14 days post-OVX, but was not significantly different from control levels at 21 days after OVX [8]. Kimmel found that OVX-induced trabecular bone loss is thought to precede cortical bone loss by up to 100 days in rats [34]. In the present study, the percentages of apoptotic and necroptotic cells were found to be higher at 8 weeks than at 4 weeks in the OVX group, likely due to the extended time course associated with the removal of matrix-bound apoptotic or necroptotic cells [35,36].As osteocytes are in the unique position of being embedded in a hard matrix, it is likely that the only cell type capable of efficiently removing dying osteocytes is osteoclasts. The time course of the disappearance of osteocytes after death may require more than 3 weeks [37]. However, the percentages of apoptotic and necroptotic cells were lower at 12 weeks than at 8 weeks in the OVX group in the present study. We speculate that this may be due to a higher bone turnover rate in the rats. An increase in osteocytes death may be partly offset by an increase in bone renewal and replacement. However, the specific signal transmission mechanism is not yet clear.

The OVX animal model has been used to study various clinical syndromes, including osteoporosis [4]. Loss of bone mass and deterioration of the bone microarchitecture have been linked to an imbalance between bone formation and bone resorption. CTX telopeptides are proteolytic fragments of type 1 collagen that form during bone resorption, and serum CTX is therefore used as the principal biomarker of bone resorption rates [38]. In accordance with other research [39], our results showed that the increased serum concentration of CTX observed at 8 weeks after OVX may be a consequence of increased resorptive activity. Measurement of BMD through DXA is a valid method for diagnosing osteoporosis [40]. In the present study, the BMD of the proximal femur was significantly lower at 12 weeks than at 0 weeks in the OVX group. The microarchitecture of Ward’s triangle was assessed via micro-CT, and the 3D reconstruction revealed that OVX dramatically reduced trabecular bone overtime. All of these results support the concept that bone loss in OVX rats mimics postmenopausal osteoporosis. However, there are some limitations to this study. 1) This study was only a pilot study of osteocyte necroptosis in OVX rats; the complicated molecular mechanisms that control necroptosis deserve further investigation. 2) These findings may be unique to the animal model, and whether they correlate with human postmenopausal osteoporosis must be ascertained in a randomized, multi-center, controlled clinical trial.

## Conclusions

In conclusion, the present study demonstrates that OVX-induced estrogen loss in the femur of female rat results in an increase in the proportion of osteocytes undergoing necroptosis in addition to apoptosis. These data confirm an important role for osteocyte necroptosis in the process of estrogen deficiency-induced bone loss in this OVX rat model. These findings may lead to an improved understanding of bone loss that occurs after clinical estrogen withdrawal and in experimental animals.

## Supporting Information

S1 Fig Areal BMD measurement in the proximal femur. (TIF)

S2 Fig Areal Ward’s triangle measurement in the proximal femur. (TIF)

S3 Fig E2 measurement. (TIF)

S1 File Blot original figures. (ZIP)

S2 File Serum E2 assay. (DOCX)
