# Supplementary figures and images for: The RIP1–RIP3 Complex Mediates Osteocyte Necroptosis after Ovariectomy in Rats
Source: PLoS One. 2016 Mar 17;11(3):e0150805. doi: 10.1371/journal.pone.0150805 (PMC4795547; doi:10.1371/journal.pone.0150805)

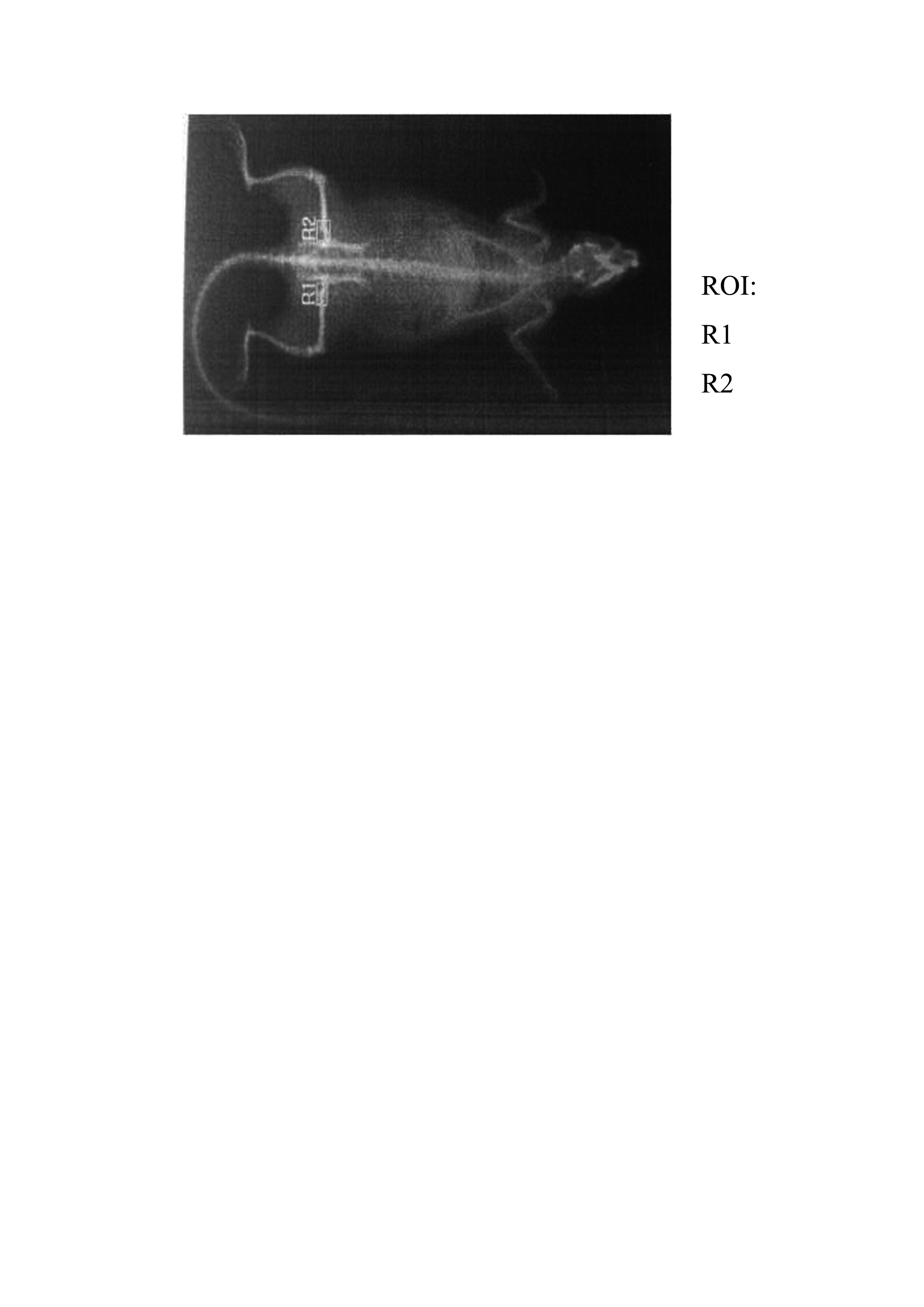

Supplement: S1 Fig — (TIF) [file pone.0150805.s001.tif]

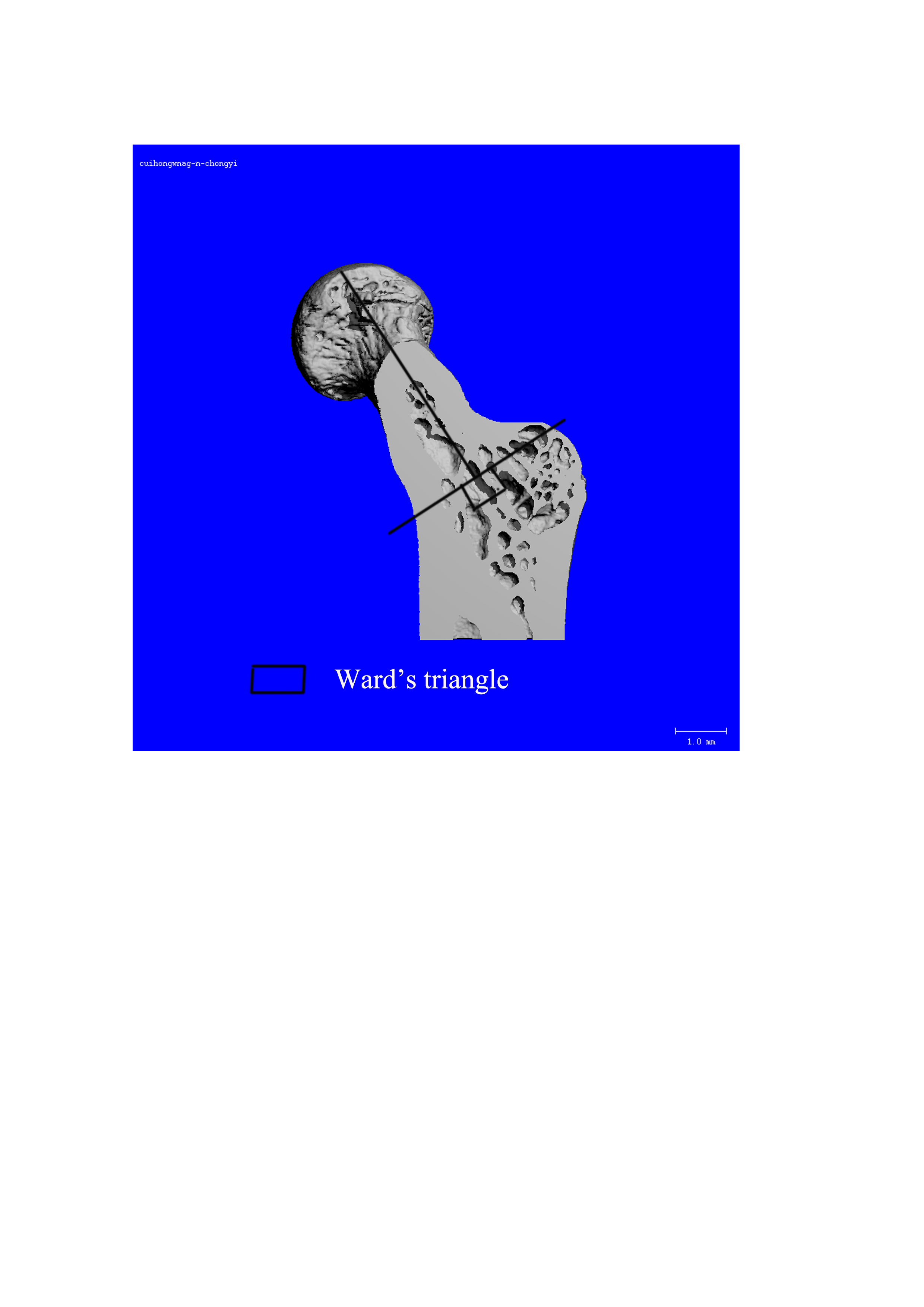

Supplement: S2 Fig — (TIF) [file pone.0150805.s002.tif]

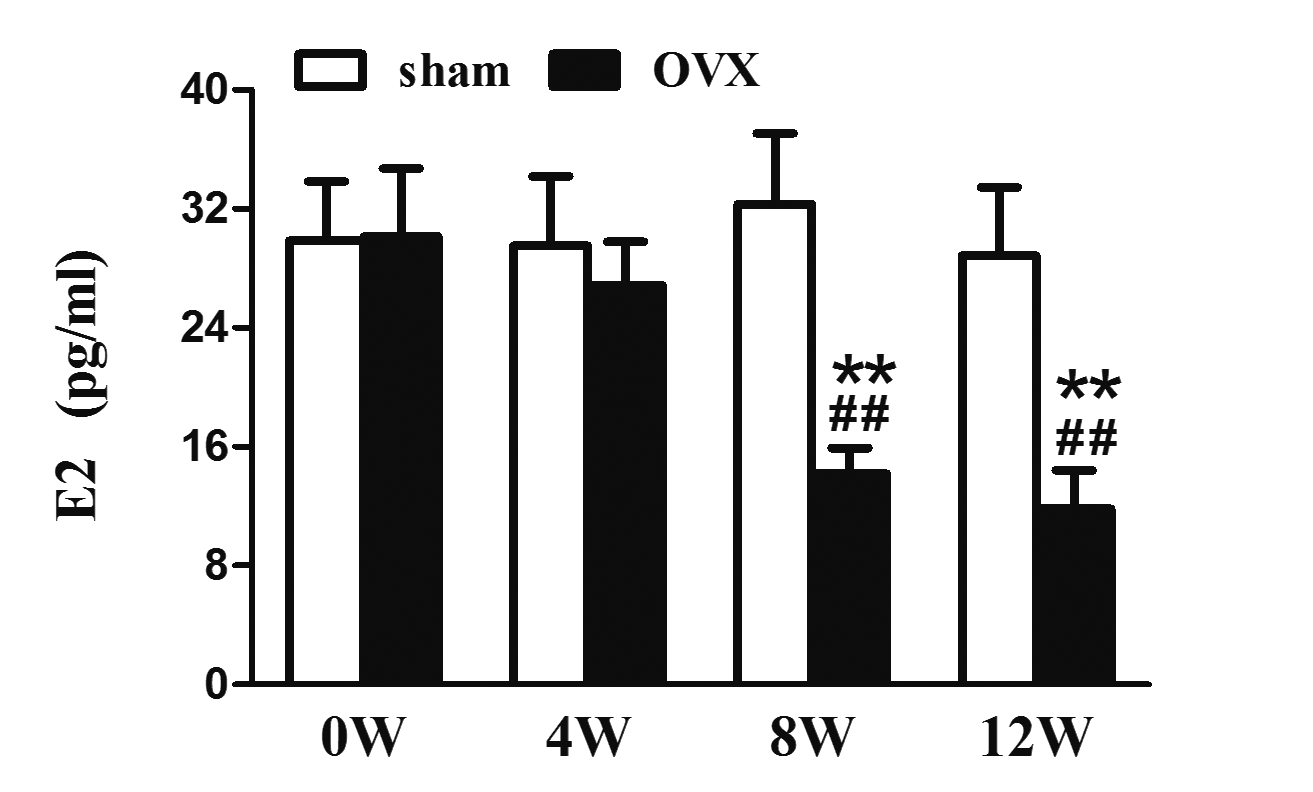

Supplement: S3 Fig — (TIF) [file pone.0150805.s003.tif]

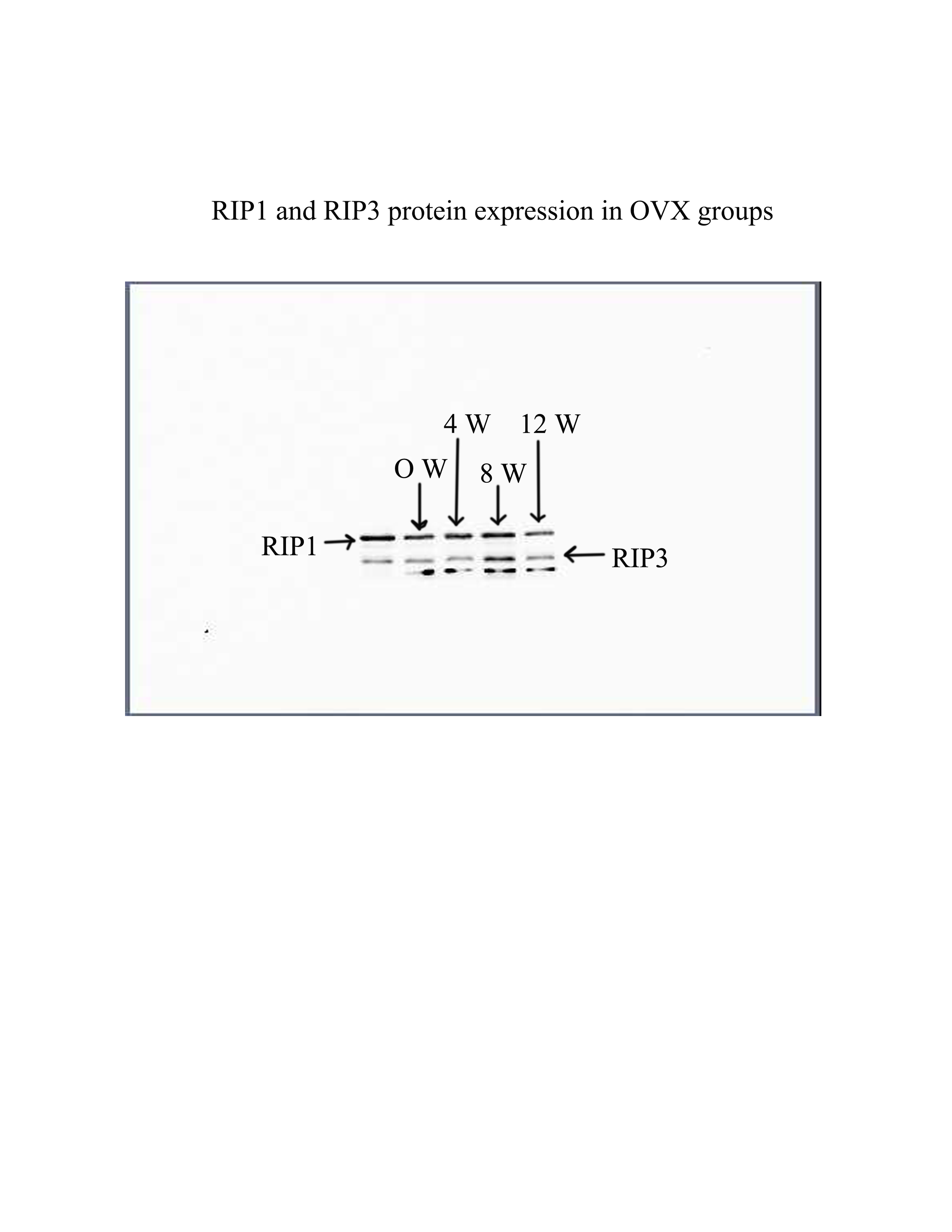

Supplement: S1 File — (ZIP) [file pone.0150805.s004.zip › blot original figures/RIP1 and RIP3 protein expression in OVX groups-labelled.tif]

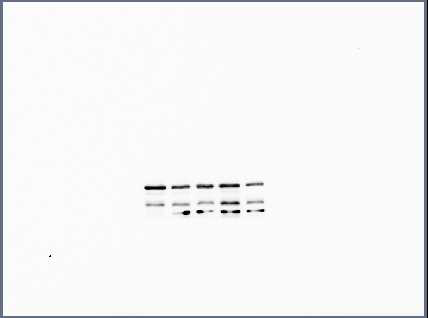

Supplement: S1 File — (ZIP) [file pone.0150805.s004.zip › blot original figures/RIP1 and RIP3 protein expression in OVX groups-original.tif]

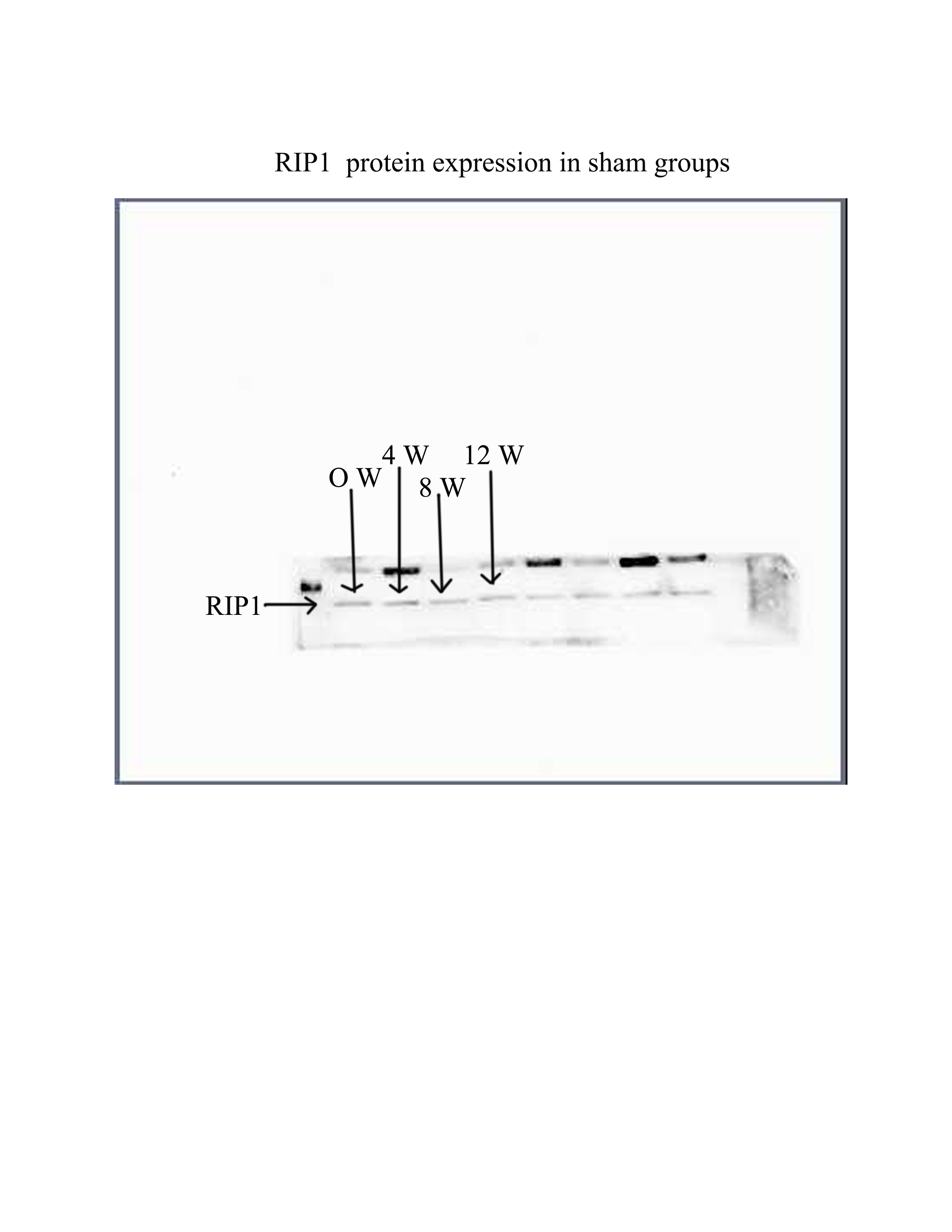

Supplement: S1 File — (ZIP) [file pone.0150805.s004.zip › blot original figures/RIP1 protein expression in sham group-labelled.tif]

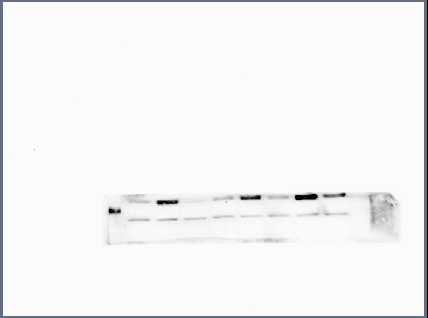

Supplement: S1 File — (ZIP) [file pone.0150805.s004.zip › blot original figures/RIP1 protein expression in sham group-original.tif]

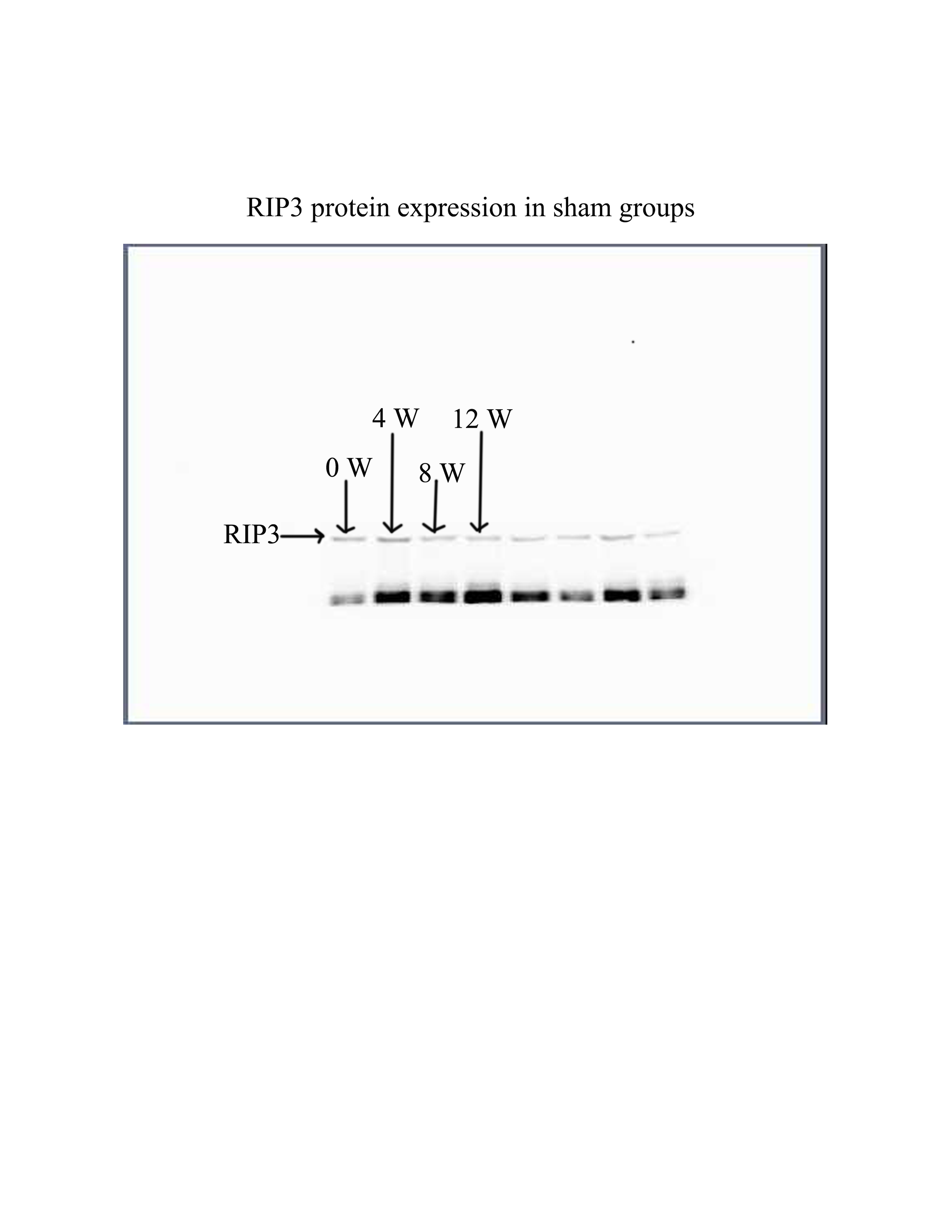

Supplement: S1 File — (ZIP) [file pone.0150805.s004.zip › blot original figures/RIP3 protein expression in sham group-labelled.tif]

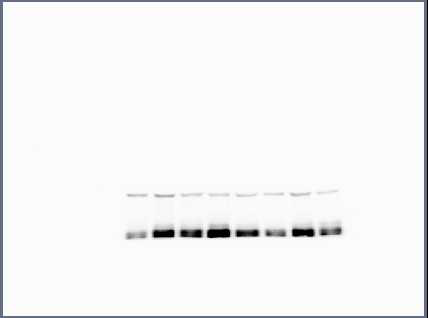

Supplement: S1 File — (ZIP) [file pone.0150805.s004.zip › blot original figures/RIP3 protein expression in sham group-original.tif]

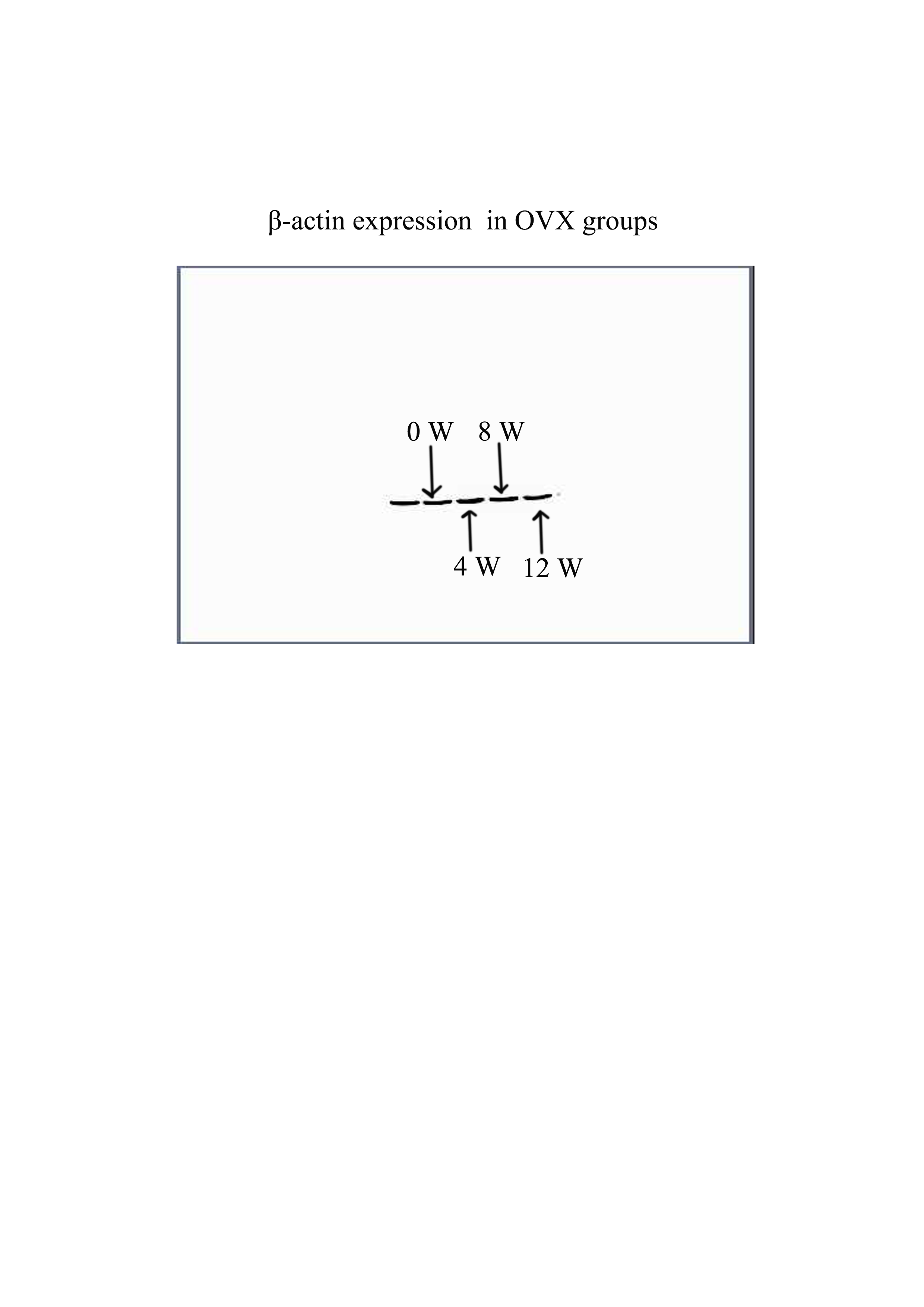

Supplement: S1 File — (ZIP) [file pone.0150805.s004.zip › blot original figures/a┬-actin expression in OVX group-labelled.tif]

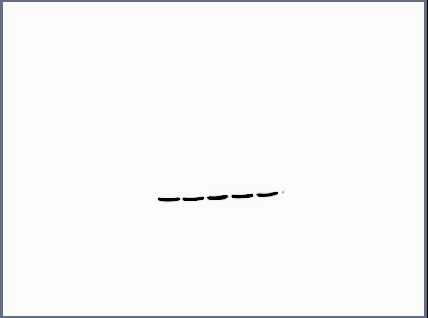

Supplement: S1 File — (ZIP) [file pone.0150805.s004.zip › blot original figures/a┬-actin expression in OVX group-original.tif]

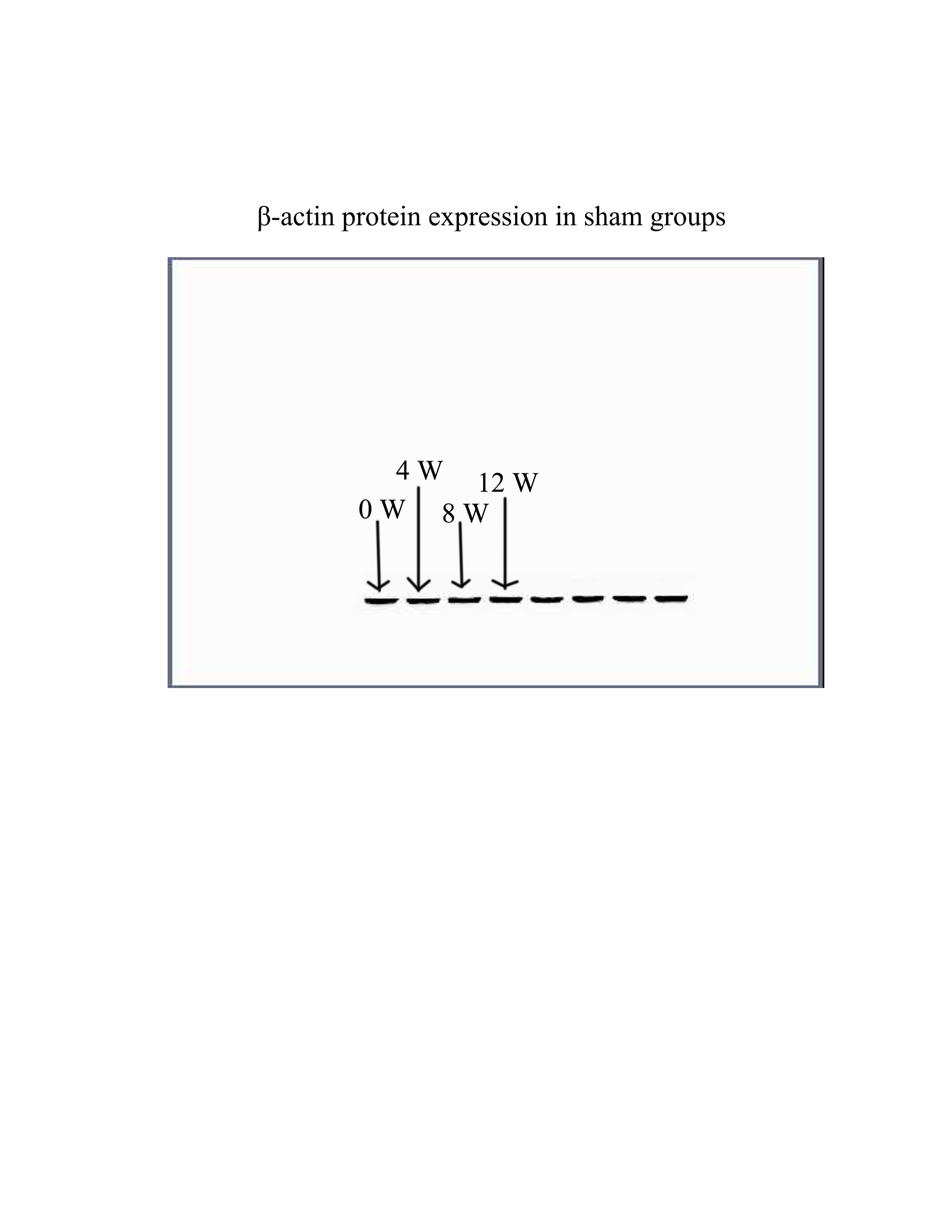

Supplement: S1 File — (ZIP) [file pone.0150805.s004.zip › blot original figures/a┬-actin expression in sham group-labelled.tif]

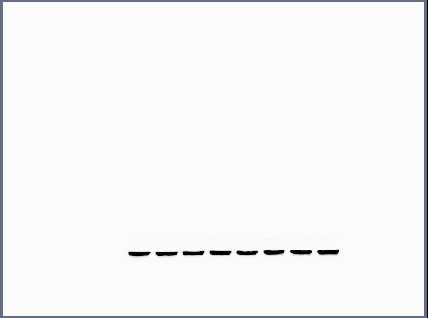

Supplement: S1 File — (ZIP) [file pone.0150805.s004.zip › blot original figures/a┬-actin expression in sham group-original.tif]
