# Supplementary material for: The RIP1–RIP3 Complex Mediates Osteocyte Necroptosis after Ovariectomy in Rats
Source: PLoS One. 2016 Mar 17;11(3):e0150805. doi: 10.1371/journal.pone.0150805 (PMC4795547; doi:10.1371/journal.pone.0150805)
Supplement: S2 File — (DOCX) [file pone.0150805.s005.docx]

**S2 File. Serum E2 assay**

Blood samples were centrifuged at 3000g for 15 min at 4°C, and the supernatant were used as serum samples. Serum E2 level (Shanghai Institute of Biological Product, Shanghai, China) was determined using commercially available ELISA kits according to the manufacturer’s protocol.

**Figure legends of E2 measurement**

The data are presented as the mean ± SEM (n=6rats/group). #p<0.05, ##p<0.01 versus the sham group at the same time point; ＊p<0.05, ＊＊p<0.01 vs the OVX group at 0 weeks. Sham, sham-operated group. OVX, ovariectomy group. W, week.
